# Burden of fatigue among adults living with HIV/AIDS attending antiretroviral therapy in Ethiopia

**DOI:** 10.1186/s12879-020-05008-4

**Published:** 2020-04-15

**Authors:** Tsiwaye Gebreyesus, Addisalem Belay, Gebretsadik Berhe, Gebremedhin Haile

**Affiliations:** 1 Department of Physiotherapy, School of Medicine, College of Health Sciences and Ayder Comprehensive Specialized Hospital, Mekelle University, P.O.Box: 1871, Mekelle, Ethiopia; 2 Department of Epidemiology, School of Public Health, College of Health Sciences and Ayder Comprehensive Specialized Hospital, Mekelle University, Mekelle, Ethiopia

**Keywords:** Fatigue, HIV/AIDS, Associated factors, Prevalence, Ethiopia

## Abstract

**Background:**

Fatigue is one of the most common and devastating Human Immuno-deficiency Virus (HIV) - related symptoms, with a varying prevalence in different study areas. In Ethiopia, there is a paucity of information on the magnitude and factors associated with fatigue among HIV/Acquired Immune Deficiency Syndrome (AIDS) patients. This may lead to under-diagnosis and eventually under-management of the symptom.

**Methods:**

Institution based cross-sectional study design was conducted among 609 HIV/AIDS patients who were selected by using a systematic random sampling method. Data were collected by using interviewer administered structured questionnaire. Level of fatigue was measured by Fatigue Severity Scale.

**Results:**

The prevalence of fatigue was found to be 51.7%. The factors associated with fatigue were: Parity [AOR = 2.01; 95% CI: 1.09–3.71], CD4 count 200–499 cells/mm3 [AOR = 2.81; 95% CI: 1.58–4.99], anemia [AOR = 4.90 95% CI: 2.40–9.97], co-morbidities [AOR = 3.65; 95% CI: 1.71–7.78], depression [AOR = 3.68 95% CI: 1.99–6.79], not being physically active [AOR = 3.20 95% CI: 1.50–6.81], clinical stage II or IV HIV [AOR = 3.11; 95% CI: 1.51–6.40] and [AOR = 4.08; 95% CI: 1.37–12.14], respectively.

**Conclusion:**

The finding of this study revealed that fatigue is a common health problem among adult People Living with HIV (PLHIV). Factors associated with fatigue included: Parity, CD4 count 200–499 cells/mm3, Clinical Stage II or IV HIV, anemia, co-morbidities, depression, and not being physically active. The health care service needs to address the predisposing factors by provision integrated care including timely detection and treatment of comorbidities, mental health problems, and promote physical activity to slow down disease progression and then reduce exposure to fatigue.

## Background

Human Immuno-deficiency Virus (HIV) is one of the most prevalent chronic health conditions, which attacks the body’s immune system and interferes with the ability to fight infection [1]. In 2017, around 36.9 million people were reported to live with HIV (PLHIV) globally, and the majority (76%) of the victims were located in sub-Saharan Africa [1, 2]. Ethiopia is one of those countries with estimated prevalence of 1.1% [3]. Despite substantial regional variations, the prevalence of HIV/AIDS in Tigray regional state was 1.2% [4].

Even though highly active antiretroviral therapy (HAART) helped patients to live longer and healthier lives, it is accompanied with numerous challenges in terms of associated symptoms and treatment side effects [5–7]. Fatigue is one of the most frequently reported symptoms by HIV/Acquired Immune Deficiency Syndrome (HIV/AIDS) patients [8, 9], which is defined as a ‘subjective sensation of weariness, increasing sense of effort, mismatch between effort expended and actual performance, or exhaustion which is not relived by additional sleep or rest’ [10].

A systematic review of 42 international studies in developed countries reported that the prevalence of fatigue among HIV/AIDS patients varied from 33 to 88% [11]. In Africa, evidence on the prevalence and related factors of HIV-related symptoms including fatigue remains unclear [8]. Studies suggest various socio-demographic [12, 13], disease and treatment related [14–16], psychosocial and personal factors [17–19] as associated factors for having fatigue.

The prevalence of fatigue among HIV/AIDS patients differs across different studies. An evidence on fatigue among HIV/AIDS patients in 2013 reported the prevalence to be between 55 and 65% [20]. Fatigue had been reported as one of the most common HIV-related symptoms that patients with HIV/AIDS experience in life [21–25].

These consequences adversely affect patients day-to-day activities and can result in poor quality of life, leading to undesirable consequence on antiretroviral therapy (ART) adherence [22]. It can also impose burden on family and community by affecting patients’ job desire and productivity level [20, 23, 24]. Studies also found out that higher fatigue level was reported by HIV-infected patients, who were single, unemployed, and under-weight [12, 20, 26, 27]. Similarly, the finding of the study from USA showed that having inadequate income had an association with higher fatigue intensity [28]. Additionally, lower educational level and urban residence are significantly associated with fatigue [8, 9].

Some studies showed that psychosocial and personal factors such as depression and psychiatric cases were found to be strong predictors of fatigue among PLHIV [11, 29, 30]. A longitudinal study in the United States of America (USA) revealed that depression had a strong relationship with fatigue report [20]. In addition, severity of insomnia had been associated with greater fatigue, and depression may contribute to both insomnia and fatigue [31]. Being physically active has also been reported as a protective factor for having fatigue among HIV-positive patients [19, 32, 33].

There is a scarcity of publications reporting the prevalence and associated factors of fatigue and in the sub-Saharan region, particularly in Ethiopia. Therefore, the aim of this study was to determine the prevalence and identify factors associated with fatigue among adults living with HIV/AIDS attending antiretroviral therapy at health facilities of Mekelle city, Tigray, Northern Ethiopia.

## Methods

### Study area and period

This study was conducted in public and private health facilities of Mekelle city, Tigray, North Ethiopia. The city has seven local administrative district (kifleketema) and one hundred sub-districts (ketena). Mekelle zone has an estimated total population of 256,000 [34]. Among the health facilities in the city, all hospitals and eight health centers provide ART service. According to the report of Tigray regional state health bureau, a total of 9662 adult ART users are accessing the service from 14 public. An institutional based cross-sectional study was conducted from April 1 to 30, 2019.

### Population, sample size and sampling procedures

The source population was all adults (Age ≥ 18) living with HIV/AIDS and those who attended ART clinic at the health facilities. Patients diagnosed with HIV/AIDS at least 30 days prior to enrollment to the study were included. Patients having a diagnosed bipolar disorders, schizophrenia, or dementia, women with known pregnancy, and patients with chronic illness condition marked by fatigue such as renal disease, cancer, multiple sclerosis, or known cardiac patients were excluded from the study. The sample size was calculated by using single population proportion formula. Since there was no similar study in the area or countries having the same status with Ethiopia, the proportion of 50% was taken to have maximum sample size. This gave a sample of 369 and by considering 10% non-response rate and 1.5 design effect; the final calculated sample size was 609. According to Tigray health bureau report, at the end of 2018, the total patients attending ART clinic in Mekelle city were 9662 with an estimated number of 3220 patient flow per month. A multistage sampling procedure was used to select study subjects and the total numbers of health facilities were selected by using a simple random sampling technique. Accordingly, 50% of the health facilities, i.e. two public hospitals, one private hospital, and four health centers were selected. The number of study units to be sampled from each facility was determined by using a proportional allocation and systematic random sampling was employed to select each study subjects. By using ART user card number, the first participant was selected by simple random sampling technique and every K^th^ interval was followed until the allocated number of study subjects for each facility was reached. The sampling fraction (K^th^) was stated as (the ART case load /sample size), every 5th ART user was recruited for each health facilities.

### Study variables and measurement tools

#### Socio-demographic factors

Gender, age, marital status, parity, educational status, occupation, residence, average monthly income, and body mass index (BMI).

#### Disease and treatment related factors

Recent cluster of differentiation 4 (CD4) count, duration since HIV confirmed, current World Health Organization (WHO) classification of HIV clinical stage, current status of anemia, current drug regimen and, current co-morbidities.

#### Psychosocial and personal factors

Depression, insomnia, physical activity, presence of physical disability.

### Measurement of variables

Burden is defined as the prevalence of fatigue. By using the Fatigue Severity Scale, a patient who scored greater than or equal to four average score was considered as having clinically significant fatigue [35]. A patient was considered as physically active if the total minutes reported by participants was greater than 150 min per week [19]. Co-morbidity was defined as the presence of one or more additional confirmed diseases or disorders concurrently occurring with HIV/AIDS (hypertension, epilepsy, opportunistic infections including tuberculosis) [36]. A participant was considered as physically disabled on self-report by the presence or absence of physical disability that had significant negative impacts on one or more major activities of daily living. Participants who scored ≥5 on ‘patient’s health questionnaire-9’ were considered as having depression. Participants whose score was > 7 on ‘Insomnia Severity Scale’ were considered to have insomnia. Body mass index is the ratio of weight to height in meters squared, less than 18.5 being underweight, 18.5–24.9 normal weight, 25–29.9 overweight, and > 30 obesity.

### Data collection tool and procedures

The tool was adopted from Fatigue Severity Scale (FSS) for measuring fatigue intensity, and Insomnia Severity Index for measuring insomnia. Data on socio-demographic, disease and treatment related characteristics, psychosocial and personal characteristics were collected with interviewer administered structured questionnaire. Data abstraction format was also used to collect data from patients’ medical record. Depression was measured by using patient’s questionnaire-9, and there was some modification in question number 9. Question number 9 ‘Thoughts that you would be better off dead or of hurting yourself in some way’ was modified to ‘Thoughts of hurting yourself in some way, if yes, how far’ then according to their response they were asked to grade it. Data were collected by seven bachelor of science degree holder nurses and supervised by two physiotherapists. Study participants who had severe fatigue were advised to visit physiotherapy department as soon as possible.

### Data quality control

In order to maintain the quality of data, the questionnaire was translated to local language (Tigrigna) and back to English to ensure its consistency. Data collectors and supervisors were trained for 1 day on the purpose of the study, details of the data collection tool, interviewing techniques, the importance of privacy and ensuring confidentiality of the respondents prior to the actual data collection. Before the data was officially collected, the questionnaire was pre-tested on 5% (31) of the total sample size among the ART users in Wukro general hospital. Based on the findings of the pre-test, changes were made, including removing the questions around viral load and some modifications in language to the insomnia and depression scales. The supervisors made routine checkups for completeness and consistency of the data. The questionnaire was reviewed and checked for completeness, accuracy and consistency by the supervisors and investigators to take timely corrective measures. The data were coded and stored in a secure, confidential area.

### Statistical analysis

Data were cleaned and entered into Epi Info version 7 and exported to Statistical Package for Social Sciences (SPSS) version 23 software for further process and analysis. Descriptive statistics was presented in the form of frequency, percentage, mean, median, range and standard deviation. Binary logistic regression model was fitted to identify factors that were significantly associated with the outcome (fatigue). Variables having *P*-value < 0.25 in the bivariate logistic regression analyses were considered as potential candidates in the final multivariable logistic regression analysis. *P*-value < 0.05 was used to declare statistical significance in the multivariable model. Multi-collinearity was checked using Variance Inflation Factor (VIF) and the overall goodness of fit of the model was checked by using Hosmer-Lemeshow goodness of fit test. Finally, the adjusted odds ratio (AOR) with its 95% confidence interval (CI) was used to determine statistically significance and depict the results.

## Results

### Descriptive statistics

#### Socio-demographic characteristics of adults living with HIV/AIDS

Among the 609 potential study participants, a total of 567 participants participated in this study, giving a response rate of 93.1%. Of the total participants, 327 (57.7%) were females with the mean age of 38 ± 10.79 years. Nearly half, 258 (45.5%) of the participants were married and 157 (27.7%) of them had children. Four hundred fifty five (80.2%) were from urban areas with median monthly household income of 1500 Ethiopian Birr (ETB). One hundred seventy two (30.3%) participants completed primary education and about 270 (47.6%) participants had healthy BMI score. (Table 1) Disease and treatment related characteristics of people living with HIV/AIDS.

**Table 1:** Socio-demographic characteristics of people living with HIV/AIDS attending ART, in Mekelle city public and private health facilities, Tigray, North Ethiopia, April 2019, (*n* = 567)

Variables	Frequency	%
**Age group**		
18–24	44	7.8
25–29	69	12.2
30–49	354	62.4
> =50	100	17.6
**Marital status**		
Single	108	19.0
Married	258	45.5
Divorced	119	21.0
Widowed	82	14.5
**Body mass index (BMI)**		
Under weight	187	33.0
Normal	270	47.6
Over weight	103	18.2
Obesity	7	1.2
**Occupation**		
House wife	125	22.0
Unemployed	52	9.3
Merchant	106	18.7
Daily laborer	121	21.3
Governmental/NGO Employed	134	23.6
Others (farmer and student)	29	5.1

More than half, 308 (54.3%) participants’ recent CD4 count was between 200 and 499 cells/mm3. Three hundred sixteen (55.7%) of the participants were in clinical stage I and 136 (24.0%) were anemic (Table 2).

**Table 2:** Disease and treatment related characteristics of people living with HIV/AIDS attending ART, in Mekelle city public and private health facilities, Tigray, North Ethiopia, April 2019, (*n* = 567)

Variables	Frequency	%
**CD4 counts**		
< 200 cells/mm3	91	16.0
200–499 cells/mm3	308	54.4
> =500cells/mm3	168	29.6
**WHO classification of HIV stage**		
Stage I	316	55.7
Stage II	103	18.2
Stage III	92	16.2
Stage IV	56	9.9
**Drug regimen**		
AZT + 3TC + NVP	115	20.3
AZT + 3TC + EFV	41	7.2
TD3 + 3TC + EFV	257	45.3
TDF + 3TC + NVP	121	21.4
ABC + DDI + LPV/R	33	5.8
**Co-morbid health conditions**		
No	438	77.2
Yes	129	22.8
**Duration since HIV confirmed**		
0–35 months	235	41.4
≥ 36	332	58.6

#### Psychosocial and personal characteristics of adult people living with HIV/AIDS

From the total respondents half of the participants, 284 (50.1%) reported clinically significant depression and 255 (45.0%) suffered from insomnia (Table 3).

**Table 3:** Psychosocial and personal factors of people living with HIV/AIDS attending ART, in Mekelle city public and private health facilities, Tigray, North Ethiopia, April 2019, (*n* = 567)

Variables	Frequency	%
**Depression**		
No	283	49.9
Yes	284	50.1
**Insomnia**		
No	312	55.0
Yes	255	45.0
**Level of physical activity**		
No	482	85.0
Yes	85	15.0
**Physical disability**		
No	554	97.7
Yes	13	2.3

#### Prevalence of fatigue among adult HIV/AIDS patients

**Fig. 1:**
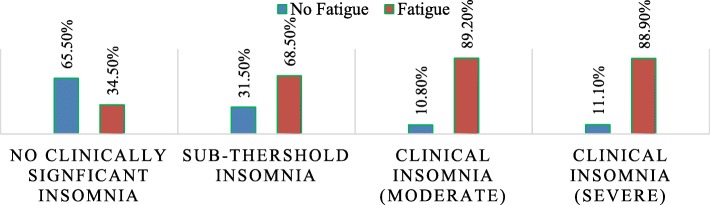
The proportion of fatigue among people living with HIV/AIDS, with different insomnia level, attending ART, in Mekelle city public and private health facilities, North Ethiopia, April 2019, (*n* = 567)

**Fig. 2:**
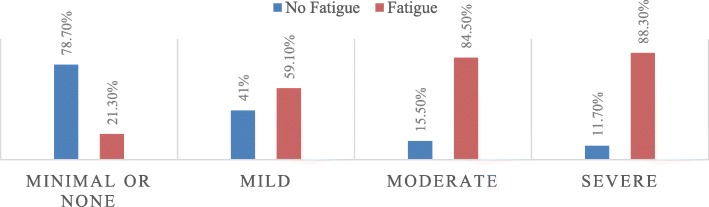
The proportion of fatigue among adults living with HIV/AIDS, with different depression level, attending ART, in Mekelle city public and private health facilities, North Ethiopia, April 2019, (*n* = 567)

Out of the 567 study participants, 293 (51.7%): 95% CI [47.4, 55.7] were found to develop fatigue. In this study, the prevalence of fatigue was higher in male participants 132 (55%), in those who were widowed 55 (67.1%), and unemployed participants 39 (75.0%). The development of fatigue was higher among anemic study participants 119 (87.5%), those with CD4 count of > 200 (67, 73.6%), those who had depression 226 (79.6%), and those participants who had physical disability (11, 84.6%) compared with their comparison groups (Table 4). Among the participants whose insomnia level was moderate, 89.2% of them had fatigue (Fig. 1), and 88.3% of the participants who had severe depression reported to have fatigue (Fig. 2).

**Table 4:** Prevalence of fatigue among adults living with HIV/AIDS attending ART, in Mekelle city public and private health facilities, Tigray, North Ethiopia, April 2019, (*n* = 567)

Socio-demographic characters		Fatigue	
		No	Yes
		Frequency (%)	Frequency (%)
Sex	Male	108 (45.0)	132 (55.0)
	Female	166 (50.8)	161 (49.2)
Age	18–24	17 (38.6)	27 (61.4)
	25–29	31 (44.9)	38 (55.1)
	30–49	184 (52.0)	170 (48.0)
	> = 50	42 (42.0)	58 (58.0)
Marital statues	Single	40 (37.0)	68 (63.0)
	Married	163 (63.2)	95 (36.8)
	Separated	44 (37.0)	75 (63.0)
	Widowed	27 (32.9)	55 (67.1)
Occupation	House wife	68 (54.4)	57 (45.6)
	Unemployed	13 (25.0)	39 (75.0)
	Merchant	52 (49.1)	54 (50.9)
	Daily laborer	62 (51.2)	59 (48.8)
	Governmental/NGO Employed	68 (50.7)	66 (49.3)
	Others (farmer and student)	11 (37.9)	18 (62.1)
Level of education	No education	66 (45.5)	79 (54.5)
	Primary school	84 (48.8)	88 (51.2)
	Secondary school	64 (47.1)	72 (52.9)
	More than Secondary	60 (52.6)	54 (47.4)
Residence	Urban	234 (51.4)	221 (48.6)
	Rural	40 (35.7)	72 (64.3)
Presence of children	No	213(52.0)	197 (48.0)
	Yes	61 (38.9)	96 (61.1)
BMI	Under weight	82 (43.9)	105 (56.1)
	Normal weight	135 (50.0)	135 (50.0)
	Over weight and obesity	57(51.8)	53 (48.2)
**Disease And Treatment Related Factors**			
CD4 count	< 200	24 (26.4)	67 (73.6)
	200–499	117 (38.0)	191(62.0)
	> = 500	133 (79.2)	35 (20.8)
WHO classification of HIV disease	Stage I	222 (48.3)	94 (51.7)
	Stage II	26 (25.2)	77 (74.8)
	Stage III	18 (19.6)	74 (80.4)
	Stage IV	8 (14.3)	48 (85.7)
Anemia status of the patient	No	257 (59.6)	174 (40.4)
	Yes	17 (12.5)	119 (87.5)
Drug regimen	AZT + 3TC + NVP	64 (55.7)	51 (44.3)
	AZT + 3TC + EFV	24 (58.5)	17 (41.5)
	TD3 + 3TC + EFV	131 (51.0)	126 (49.0)
	TDF + 3TC + NVP	36 (29.8)	85 (70.2)
	ABC + DDI + LPV/R	19 (57.6)	14 (42.4)
Co-morbid health conditions	No	256(58.4)	182(41.6)
	Yes	18(14.0)	111(86.0)
Duration since HIV confirmed			
	0–35 months	87 (37.0)	148 (63.0)
	≥ 36	187 (56.3)	145 (43.7)
**Psychosocial and personal factors**			
Depression	No	216 (76.3)	67 (23.7)
	Yes	58 (20.4)	226 (79.6)
Insomnia	No	213 (68.3)	99 (31.7)
	Yes	61 (23.9)	194 (76.1)
Physical exercise	No	211 (43.8)	271 (56.2)
	Yes	63 (74.1)	22 (25.9)
Presence of any physical disability	No	272 (49.1)	282 (50.9)
	Yes	2 (15.4)	11 (84.6)

#### Factors associated with fatigue among people living with HIV/AIDS

In the bivariate regression analysis, fatigue was significantly (*p* <  0.25) associated with sex, age, marital status, parity, occupation, residence, monthly income, BMI, CD4 cell count, total duration since HIV confirmed, clinical stage, anemia, drug regimen, co-morbidity, depression, insomnia, and physical activity. Nevertheless, in the multivariate regression analysis, fatigue was significantly (*p* <  0.05) associated with parity [AOR = 2.01; 95% CI: 1.09–3.71], CD4 cell count [AOR = 2.81; 95% CI: 1.58–4.99], anemia [AOR = 4.90 95% CI: 2.40–9.97], current WHO classification of HIV disease [AOR = 3.11; 95% CI: 1.51–6.40], depression [AOR = 3.68 95% CI: 1.99–6.79], and co-morbidities [AOR = 3.65; 95% CI: 1.71–7.78] (Table 5).

**Table 5:** Factors associated with fatigue among adults living with HIV/AIDS attending ART, in Mekelle city public and private health facilities, Tigray, North Ethiopia, April 2019, (*n* = 567)

	Yes Frequency (%)	Presence of fatigue				AOR(95%CI)
		No Frequency (%)	Crude odds ratio (COR) (95%CI)]	P-V	P-V	
**Presence of children**						
Yes	96(61.1%)	61(38.9%)	1.70(1.17–2.47)	**< 0.005**^**a**^	**0.025**^**b**^	**2.01(1.09–3.71)**^**b**^
No	197(48.0%)	213 (52.0%)	1.000			
**CD4 count**						
< 200	67(73.6%)	24(26.4%)	10.608(5.841–19.266)	**< 0.001**^**a**^	0.09	2.14(0.89–5.10)
200–499	191(62.0%)	117(38.0%)	6.203(4.004–9.610)	**< 0.001**^**a**^	**< 0.001**^**b**^	**2.81(1.58–4.99)**^**b**^
> =500	35(20.8%)	133(79.2%)	1.000			
**WHO classification of HIV disease**						
stage I	94(29.7%)	222(70.3%	1.000			
Stage II	77(74.8%)	26(25.2%)	6.994(4.218–11.598)	**< 0.001**^**a**^	**0.002**^**b**^	**3.11(1.51–6.40)**^**b**^
Stage III	74(80.4%)	18(19.6%)	9.709(0.207–1.179)	**< 0.001**^**a**^	0.063	2.13(0.96–4.76)
Stage IV	48(85.7%)	8(14.3%)	14.170(6.454–31.110)	**< 0.001**^**a**^	**0.011**^**a**^	**4.08(1.37–12.14)**^**b**^
**Anemia status of the patient**						
Yes	119(87.5%)	17(12.5%)	10.339(6.005–17.802)	**< 0.001**^**a**^	**< 0.001**^**b**^	**4.90 (2.40–9.97)**^**b**^
No	174(40.4%)	257(59.6%)	1.000			
**Co-morbid health conditions**						
Yes	111(86.0%)	18(14.0%)	8.674(5.090–14.781)	**< 0.001**^**a**^	**0.001**^**b**^	**3.65(1.71–7.78)**^**b**^
No	182(41.6%)	256(58.4%)	1.000			
**Depression**						
Yes	226(79.6%)	58(20.4%)	12.562(8.438–18.702)	**< 0.001**^**a**^	**< 0.001**^**b**^	**3.68(1.99–6.79)**^**b**^
No	67(23.7%)	216(76.3%)	1.000			
**Physical activities**						
Yes	22(25.9%)	63(74.1%)	1.000			
No	271(56.2%)	211(43.8%)	3.678(2.192–6.172)	**< 0.001**^**a**^	**0.003**^**b**^	**3.20(1.50–6.81)**^**b**^

NB: COR = crude odds ratio, AOR = adjusted odds ratio, ^a^ = significant association (on bivariate), ^b^ = significant association (on multivariate), 1.000 = Reference

## Discussion

This study mainly investigated the prevalence and factors associated with fatigue among HIV patients attending ART in public and private health facilities in Mekelle city, Ethiopia. In the current study, the prevalence of fatigue was found to be 51.7%: 95% CI [47.4, 55.7]. This result is comparable with the finding of a study done in South Africa, 55% [8], and USA, 54% [37]. This consistent result can be explained by the use of large sample size, similarities in study population and similarities in eligibility criteria.

On the other hand the prevalence in our study was lower than the finding of other studies done in South Africa, 66.7% [9], United Kingdom (UK), 65.1% [17], Uganda (61%) [38], and China, 86.8% [31]. This discrepancy could be due to difference in study criteria, for example, the study in South Africa used study-subjects who were extremely ill and the other two studies (Uganda and the UK) used participants who developed at least two HIV-related symptoms, which may increase the prevalence of fatigue. The other possible reason can be the difference in assessment tools. The study from South Africa used the revised Sign and Symptom Checklist for persons with HIV disease, the study from the UK used Chalder Fatigue Scale, and Short Form of Memorial Symptom Assessment Scale was used in Uganda. The first two studies reported the prevalence of fatigue, whereas this study reported clinically significant fatigue level (FSS > 4). Differences in data collection and sampling method could also explain the discrepancy in our results. The study that was done in South Africa used convenience sampling and the study that was conducted in China used case report forms. The study from UK used self-administered questionnaire in which case the investigators did not have information on those patients who failed to fill the questionnaire, but there was possibility that patients with fatigue were more likely to participate, which may lead to over reporting the prevalence.

Moreover, the finding of this study is relatively higher than that of the study done in UK, which reported the prevalence of fatigue to be 28% [39]. This could be due to the reason that the study done in the UK considered only severe fatigue reports to categorize as positive for fatigue, but the current study considered both moderate and severe fatigue. The higher rate of fatigue reported in HIV/AIDS patients indicate that fatigue is important comorbidity and requires adequate attention by health care providers.

In the current study, participants who had children were two times more likely to develop fatigue when compared to those who did not have children. Similar association was found from studies done in South Africa, USA, and France [8, 29, 34] which reported that patients who had children had a higher risk of developing fatigue. This result may be attributed to the additional work load and responsibilities that came with having children in terms of financial and social burdens.

In the present study, participants whose CD4 count ranged from 200 to 499 had approximately three times higher risk for developing fatigue compared to those whose CD4 count was > 500. On the other hand, a study conducted in USA revealed that patients whose CD4 count was > 200 had a high risk of developing fatigue [16]. The observed discrepancy might be due to a higher number of participants (50.3%) in the current study with CD4 count 200–499. Another possible reason could be those patients with low CD4 count may have experienced additional opportunistic infections which might have contributed to fatigue.

Stage of disease was associated with fatigue. Our finding showed that patients staged with the current WHO classification of HIV/AIDS stage II and IV were significantly associated with reported fatigue when compared with stage I, which was supported by a study done in France [34]. The reduction of CD4 count and presence of opportunistic infections in the later stages of HIV/AIDS may explain why patients may have additional fatigue intensity. This implies the health care service should provide optimum care to HIV/AIDS patients in stage II and IV to relieve them from fatigue.

This study revealed that HIV-patients who had other co-morbidities were four times more likely to develop fatigue than those who did not have co-morbidities. This finding is consistent with the study which was done in USA [40]. This could be due to the double burden of symptoms of HIV and other concurrently occurring health conditions which might have caused fatigue by them- selves.

In the current study, participants who did not perform mild to moderate physical exercise on a regular basis had an approximately four times higher risk of developing fatigue than those who were engaged in physical activity. Similarly, a study done in France reported that being physically active was significantly associated with reduced perceived physical fatigue [34]. The possible relationship might be due to the positive impact of physical exercise on the quality of life of HIV-positive patients in reducing acute fatigue, muscular weakness or pain that they experience. Moreover, physical activities could have positive effects on mood, which is believed to increase a positive influence on the reduction of perceived fatigue [41]. This might be due to the positive impact of physical exercise on the quality of life of HIV-positive patients in reducing acute fatigue, muscular weakness or pain that they experience.

The finding of the present study indicated that patients with depression were about four times more likely to report fatigue compared with patients without depression. The finding is in line with the studies done in USA [14, 23, 35]. Even though it is difficult to predict a cause and effect relationship; depression may lead to fatigue, and in turn the inability to carry out normal activities due to fatigue or having HIV/AIDS by itself may lead to depression.

In our study, participants who had anemia had approximately five times higher risk for developing fatigue than those without anemia which is in line with the studies done in the USA [38] and China [31]. Anemia is a well-known reason for fatigue. The reduction in red blood cells count below a normal level might force the body to compensate in a number of ways including redistribution of blood in order to give more oxygen to critical organs such as the brain. This redistribution may contribute to fatigue.

### Limitation of the study

Limitations of this study include the lack of information captured about study participant use of antidepressant and sleeping pills, which could have influenced the scores of ‘Patients health questionaire-9’ and ‘Insomnia Severity Scale’. Moreover, as most of the previous research was done in developed countries, it was difficult to compare the result of this study with other studies in the Sub-Saharan region. The use of secondary source can also be mentioned as a limitation of this study. Hence, further longitudinal studies in the area are needed to be conducted.

## Conclusions

The finding of this study revealed that fatigue is a common health problem among adult PLHIV. Factors associated with fatigue included: Parity, CD4 count 200–499 cells/mm3, Clinical Stage II or IV HIV, anemia, co-morbidities, depression, and not being physically active. The health care service needs to address the predisposing factors by provision integrated care including timely detection and treatment of comorbidities, mental health problems, and promote physical activity to slow down disease progression and then reduce exposure to fatigue. Authorities and health care providers are recommended to develop preventive strategies and treatment guidelines to determine practical interventions for reducing fatigue and targeting the associated factors such as depression and anemia. Further longitudinal studies in the area are needed to be conducted.

## Data Availability

Additional data could be obtained from the corresponding author upon formal request.

## Abbreviations

HIV: Human Immuno-deficiency Virus
PLHIV: People Living with HIV/AIDS
AIDS: Acquired Immune Deficiency Syndrome
HAART: Highly active antiretroviral therapy
ART: Antiretroviral Therapy
USA: United States of America
UK: United Kingdom
BMI: Body Mass Index
CD4: cluster of differentiation 4
WHO: World Health Organization
FSS: Fatigue Severity Scale
VIF: Variance Inflation Factor
SPSS: Statistical Package for Social Sciences
AOR: Adjusted Odds Ratio
CI: Confidence Interval
ETB: Ethiopian Birr
MU/CHS: Mekelle University/College of Health Sciences
